# Computational Study of Thermal Comfort and Reduction of CO_2_ Levels inside a Classroom

**DOI:** 10.3390/ijerph19052956

**Published:** 2022-03-03

**Authors:** Guillermo Efren Ovando-Chacon, Abelardo Rodríguez-León, Sandy Luz Ovando-Chacon, Martín Hernández-Ordoñez, Mario Díaz-González, Felipe de Jesús Pozos-Texon

**Affiliations:** 1 Tecnológico Nacional de México, Instituto Tecnológico de Veracruz, Calzada Miguel Ángel de Quevedo 2779, Veracruz 91860, Mexico; guillermo.oc@veracruz.tecnm.mx (G.E.O.-C.); martin.ho@veracruz.tecnm.mx (M.H.-O.); mario.dg@veracruz.tecnm.mx (M.D.-G.); felipe.pt@veracruz.tecnm.mx (F.d.J.P.-T.); 2 Tecnológico Nacional de México, Instituto Tecnológico de Tuxtla Gutiérrez, Carretera Panamericana km 1080, Tuxtla Gutierrez 29000, Mexico

**Keywords:** thermal comfort, CO_2_ reduction, ventilation, COVID-19, classroom, turbulent convection, CFD simulations

## Abstract

Due to the current COVID-19 pandemic, guaranteeing thermal comfort and low CO_2_ levels in classrooms through efficient ventilation has become vitally important. This study presents three-dimensional simulations based on computational fluid dynamics of airflow inside an air-conditioned classroom located in Veracruz, Mexico. The analysis included various positions of an air extractor, Reynolds numbers up to 3.5 × 10^4^, four different concentrations of pollutant sources, and three different times of the day. The simulations produced velocity, air temperature, and CO_2_ concentrations fields, and we calculated average air temperatures, average CO_2_ concentrations, and overall ventilation effectiveness. Our results revealed an optimal extractor position and Reynolds number conducive to thermal comfort and low CO_2_ levels due to an adequate ventilation configuration. At high pollutant concentrations, it is necessary to reduce the number of students in the classroom to achieve safe CO_2_ levels.

## 1. Introduction

Over the past twenty years, in the context of climate change associated with the use of energy obtained from fossil fuels, many studies on non-isothermal flows in spaces have addressed the problem of how to achieve thermal comfort while optimizing energy use. However, these studies should also take into account air quality, especially in closed spaces where large numbers of people are present, often involving high CO_2_ values (above 1000 ppm) as a product of human activity and poor air circulation. Before the SARS-CoV-2 pandemic, millions of students around the world attended classrooms requiring energy-efficient ventilation systems that guaranteed thermal comfort and low levels of pollutants, such as CO_2_. The above was necessary since comfortable and healthy conditions in closed spaces, such as classrooms, favor the learning process because they prevent inattention or drowsiness. However, the pandemic has made it more imperative than ever to ensure low levels of CO_2_ since this parameter can serve as an indicator of the risk of contagion among the academic community in schools, universities, and other higher education institutions. The problem is more serious when high CO_2_ concentrations occur in closed spaces, such as classrooms, where students and teachers are together for long periods [1,2,3,4], putting their health at risk and significantly reducing student achievement [1].

Therefore, during the last decade, several studies indirectly [5,6,7,8,9] or directly [1,10,11,12] focused on the problem of pollutant concentration and thermal comfort in closed spaces. All of the research focusing indirectly on excess CO_2_ (or other contaminants) approaches the problem by using numerical studies in cavities, namely computational fluid dynamics (CFD) simulations [5,6,7,9,13]. Although these are excellent theoretical works, these studies are characterized by not having a direct application to a problem in a defined space in the real world. Research directly focusing on excess CO_2_ analyzes existing closed spaces for human use in the real world. Some of these studies represent strictly applied research, such as [10], in which the effect of CO_2_ sensors was studied to improve the performance of forced convection ventilation systems. On the other hand, studies such as [11,14,15,16,17,18,19,20,21] were conducted in real spaces, such as industrial buildings for welding processes, hospitals, computer laboratories, offices, mosques, kitchens, buildings, dorm rooms, or schools.

Other studies have focused explicitly on excess CO_2_ in educational spaces [1,2,3,4,12,22,23,24]. Of these, several experimental studies, such as [1,2,3,4], measure CO_2_ concentrations in academic settings using different methods. A remarkable case is [2]; in addition to controlling contamination by CO_2_, the authors sought specifically to reduce the transmission of SARS-CoV-2 in the educational environments using experimental measurements to infer the behavior of the fluid in the closed space and established a correlation between ventilation aimed at improving air quality and the decrease in contagions. A detailed study of airflow behavior in a closed space using CFD allows a better understanding of the phenomenon to achieve good air circulation that reduces CO_2_ and, incidentally, contributes to lowering the SARS-CoV-2 contagion rate. Similarly, ref. [25] conducted a study to understand ozone distribution in closed spaces using CFD simulations in turbulent flow regimes to predict ozone levels in a SARS-CoV-2-disinfected area.

Until now, there are very few CFD studies in schools where thermal comfort is analyzed considering a large number of students; in [26,27], classrooms without air conditioning and with open windows were studied. On the other hand, other CFD studies inside spaces have considered the transport of particles to study the transmission of contagious diseases in schools [28,29] and hospitals [30,31,32,33].

Most of the numerical studies on indoor CO_2_ concentration published so far have been based on simple geometry and a pollutant source in the walls, and only a few have considered a large number of people inside the space. On the other hand, none of these studies have considered the flux of heat through closed windows of an air-conditioned classroom. Due to the above, it is of great importance to determine how the position of a ceiling extractor affects the thermal comfort conditions and the CO_2_ values in an air-conditioned classroom with students. The purpose of the present study is to perform numerical simulations in a three-dimensional geometry; to know the effects of extractor position and Reynolds numbers on the ventilation; and to find a configuration that guarantees thermal comfort and low CO_2_ levels in an air-conditioned classroom for various concentrations of pollutant sources and different heat fluxes. In the analyzed classroom, thirty students and one teacher were considered. In Mexico and especially in places with a hot climate, such as the port of Veracruz, many public schools have poorly designed air-conditioning systems. This causes a deficient removal of CO_2_, and for this reason, it is necessary to know the specific flow pattern inside the classrooms to find the proper arrangement of the equipment that guarantees better air quality.

## 2. Methodology

### 2.1. Physical Model

**Figure 1:**
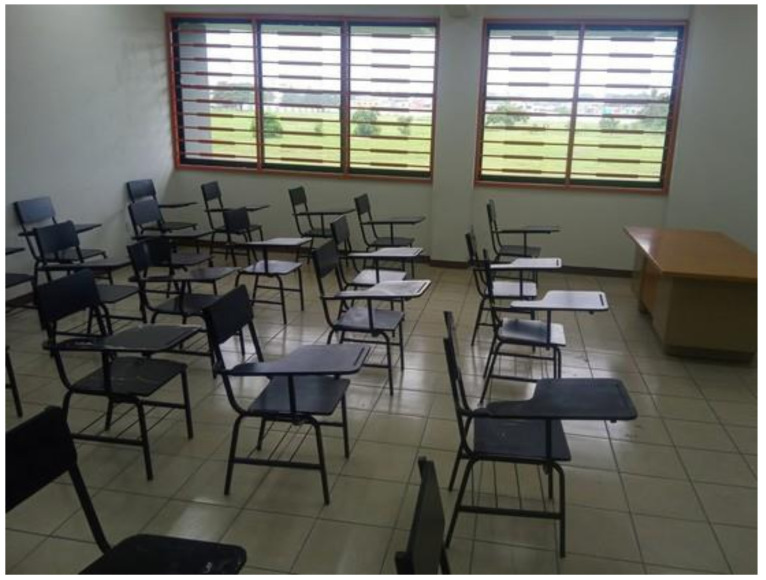
Classroom analyzed in building “X” of the Tecnológico Nacional de México, Campus Veracruz, located in the port of Veracruz, Mexico.

**Figure 2:**
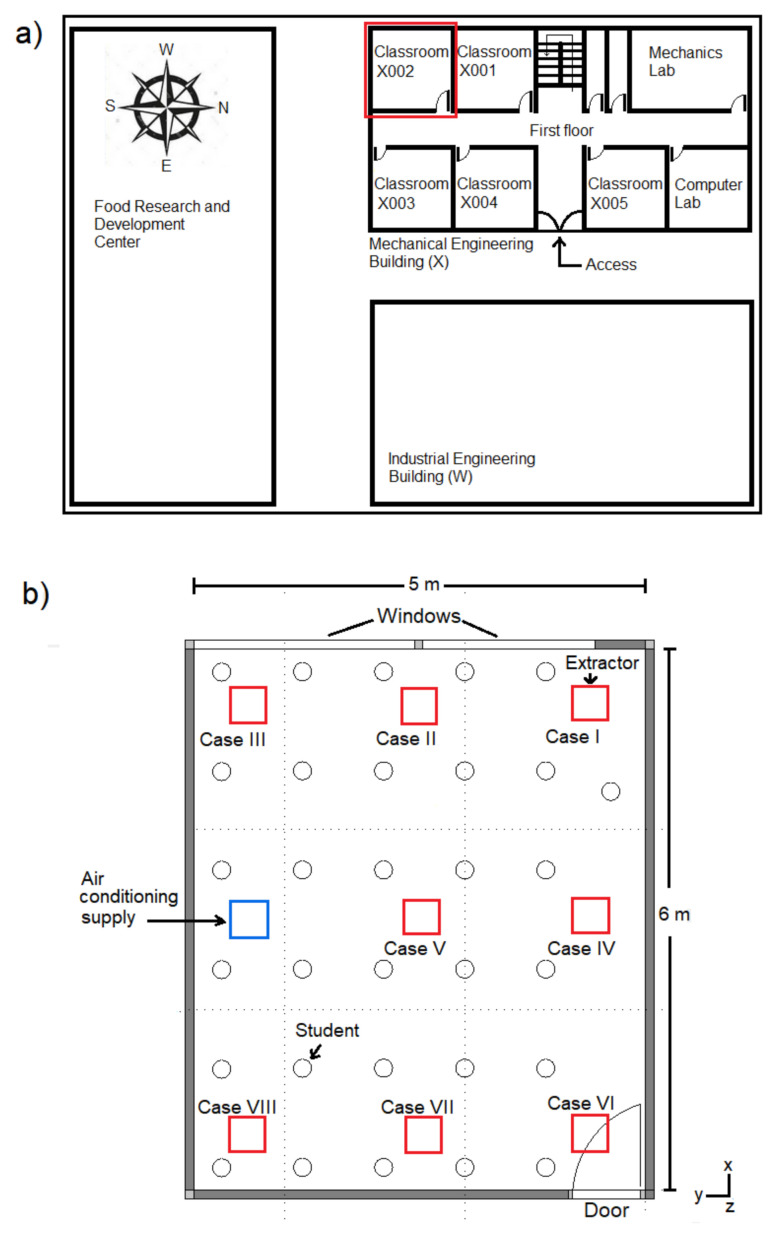
Plan view: (**a**) Building “X”. (**b**) Classroom “X002”. The red boxes indicate the different positions of the air extractor considered in this study.

Figure 1 shows the undergraduate classroom (X002) analyzed in the present study. The classroom is located in building “X” of the Department of Mechanical Engineering of the Tecnológico Nacional de México, Campus Veracruz, located in the port of Veracruz, México, where warm climate prevails. The study considered 30 students and one teacher inside the space. The west wall has two closed windows, one measuring 2.2 m × 2 m and the other 1.5 m × 2 m, through which there are heat gains inside the room, mainly after 11:00 a.m., due to solar radiation. It is important to note that the windows cannot be opened, so they always remain closed. The east wall was considered to be at a constant temperature of 25 °C. The south wall is exposed to the outside and is in front of a tall building that shades it most of the day; meanwhile, the north wall adjoins another classroom. The south and north walls are considered adiabatic. Air-conditioning equipment (440–1150 m^3^/h) was available in the classroom at the top center of the ceiling and near the south wall. Figure 2a shows a plan view of the classroom, building “X”, and its surroundings. The classroom’s door is in the west wall, which adjoins a common hall that connects with other classrooms and the rest of the building; the door is usually closed during classes. On the other hand, the main doors of the building are typically locked during the day, opening and closing only to allow teachers and students to access or exit the building; as a consequence, the air inside the building, especially in the most crowded classrooms, is very poor. Therefore, it is necessary to propose improvements to reduce CO_2_ concentration in the classrooms. In the present study, three-dimensional numerical simulations of airflow and energy and mass transport were carried out inside the classroom previously described. The analysis was performed for different Reynolds numbers (Re) in the range of 1000 to 35,000, with CO_2_ sources (C_s_) from 35,000 ppm to 42,500 ppm, generated by the students and the teacher and distributed in the classroom. The heat fluxes imposed through the classroom windows were 95.8 W/m^2^, 326.6 W/m^2^, and 147.6 W/m^2^, which correspond to three different hours of the day: 11:30 a.m., 3:30 p.m., and 6:30 p.m., respectively. An air extractor was installed to reduce CO_2_ concentration levels inside the room, and the effect of eight different positions of this device, as shown in Figure 2b, was analyzed. The simulations were performed for inlet velocities in the range of 0.21–1.46 m/s, with inlet temperature of 22 °C and CO_2_ concentration of 340 ppm. Persons were considered inside the room with a heat flux of 75 W. The study considered a production of CO_2_ per occupant in the range of 9.456 × 10^−6^ kg/s to 1.20 2 × 10^−5^ kg/s.

The analyzed classroom is located in a recently created building where undergraduate classes took place from January 2019 to 23 March 2020, when classes were interrupted as an official health measure mandated by the federal government of Mexico to reduce the spread of COVID-19. The classrooms located in this building lack an efficient ventilation system. In addition, due to the warm climate in the city of Veracruz, the building and the classrooms are fully air-conditioned. Therefore, the doors and windows remain closed all day. Consequently, CO_2_ concentrations are high at almost all times. In June 2019, different measurements were taken inside the classroom, observing that during the class that started at 11:00 a.m., CO_2_ concentration increased rapidly above 10:00 ppm, while pollutant concentrations decreased below 1000 ppm in the class that started at 7:00 p.m. Due to the fact that all the classes have a duration of 1 h, the study was carried out in the middle of the class that started at 11:00 a.m., that is, at 11:30 a.m., and in the middle of the previous class at 7:00 p.m., that is, at 6:30 p.m. On the other hand, the measurements indicated that the maximum heat flux through the closed windows of the classroom occurred during the 3:00 pm class; therefore, the study was carried out in the middle of that class, that is, at 3:30 p.m.

To solve the problem of high concentrations of CO_2_, the thermal behavior of the air flow and the concentration of the pollutant in the classroom were analyzed through numerical simulations for different conditions of heat transfer through the closed windows, which correspond to three different hours of the day. The simulations provide velocity, temperature, and CO_2_ concentration fields, average temperatures and CO_2_ concentrations in the classroom, and overall ventilation effectiveness for air temperature distribution and overall ventilation effectiveness for CO_2_ removal.

### 2.2. Governing Equations

Turbulent flow, CO_2_ distribution, and temperature inside a classroom subject to thermal loads and sources of pollutant are described by Navier–Stokes, mass transport, energy, and turbulence equations. The steady-state approach was used successfully by other researchers to study the concentration of CO_2_ in indoor spaces [5,6,7,34]. The steady-state governing equations used in this work are:

(1) $$\frac{\partial \left({\rho u}_{i}u_{j}\right)}{\partial x_{j}}=-\frac{\partial p}{\partial x_{i}}+\frac{\partial }{\partial x_{j}}\left[\mu \left(\frac{\partial u_{i}}{\partial x_{j}}+\frac{\partial u_{j}}{\partial x_{i}}\right)-\bar{{\rho u}_{i}^{´}u_{j}^{´}}\right]+F_{i}$$

(2) $$\frac{\partial \left({\rho u}_{i}\right)}{\partial x_{i}}=0$$

(3) $$\frac{\partial \left({\rho C}_{p}u_{j}T\right)}{\partial x_{j}}=\frac{\partial }{\partial x_{j}}\left[\lambda \frac{\partial T}{\partial x_{j}}-{\rho C}_{p}\bar{u_{i}^{´}T´}\right]$$

(4) $$\frac{\partial \left(u_{j}C\right)}{\partial x_{j}}=\frac{\partial }{\partial x_{j}}\left[D\frac{\partial C}{\partial x_{j}}-\bar{u_{i}^{´}C´}\right]$$

where u_i_ is the *i* component of the velocity, x_i_ is the i component of the spatial coordinate, ρ is the density, p is the pressure, µ is the dynamic viscosity, F_i_ is the i component of the body force, T is the temperature, C_p_ is the specific heat, λ is the thermal conductivity, C is the concentration, and D is the diffusion coefficient. The properties of the air–CO_2_ mixture are shown in Table 1.

In Equations (1)–(4), the Reynolds stress tensor, turbulent heat flux, and turbulent mass flow can be approximated by:

(5) $$\bar{{\rho u}_{i}^{´}u_{j}^{´}}=-\mu_{t}\left[\frac{\partial u_{i}}{\partial x_{j}}+\frac{\partial u_{j}}{\partial x_{i}}\right]+\frac{2}{3}{\rho K\delta }_{\mathrm{ij}}$$

(6) $$\rho \bar{u_{i}^{´}T´}=-\frac{\mu_{t}}{{\mathrm{Pr}}_{t}}\frac{\partial T}{\partial x_{j}}$$

(7) $$\bar{u_{i}^{´}C´}=-\frac{\mu_{t}}{{\mathrm{Sc}}_{t}}\frac{\partial C}{\partial x_{j}}$$

where Pr_t_ is the turbulent Prandtl number, and Sc_t_ is the turbulent Schmidt number. On the other hand, the body force can be described as follows:

(8) $$F_{i}={\rho g\delta }_{2j}\left[\beta_{T}\left(T-T_{\mathrm{ref}}\right)+\beta_{C}\left(C-C_{\mathrm{ref}}\right)\right]$$

The coefficients of thermal expansion β_T_ and of concentration β_C_ are calculated by β_T_ = 1/T_0_ y β_C_ = 1/C_0_, where T_0_ = 296 K and C_0_ = 340 ppm; g is the gravity acceleration. The turbulent kinetic energy K and the turbulent kinetic energy dissipation ε are calculated by:

(9) $$\frac{\partial \left({\rho u}_{i}K\right)}{\partial x_{i}}=\frac{\partial }{\partial x_{i}}\left[\left(\mu +\frac{\mu_{t}}{\sigma_{K}}\right)\frac{\partial K}{\partial x_{i}}\right]+P_{K}+G_{K}-\rho \varepsilon$$

(10) $$\frac{\partial \left({\rho u}_{i}\epsilon \right)}{\partial x_{i}}=\frac{\partial }{\partial x_{i}}\left[\left(\mu +\frac{\mu_{t}}{\sigma_{\varepsilon }}\right)\frac{\partial \varepsilon }{\partial x_{i}}\right]+C_{\epsilon 1}\frac{\epsilon }{K}\left(P_{K}+C_{\epsilon 3}G_{K}\right)-C_{\epsilon 2}\rho \frac{\varepsilon^{2}}{K}$$

In the previous equations, P_K_ is the turbulent kinetic energy production rate, G_K_ is the generation or destruction of turbulence due to fluctuations in body forces, and µ_t_ is the turbulent viscosity. The coefficients values for the turbulence model are C_ε1_ = 1.44, C_ε2_ = 1.92, C_ε3_ = tanh|u_3_/u_1_|, σ_k_ = 1.0, and σ_ε_ = 1.3. P_K_ and G_K_ can be calculated as follow:

(11) $$P_{K}=\mu_{t}\left(\frac{\partial u_{i}}{\partial x_{j}}+\frac{\partial u_{j}}{\partial x_{i}}\right)\frac{\partial u_{i}}{\partial x_{j}}$$

(12) $$G_{K}=-\beta_{T}g\frac{\mu_{t}}{\sigma_{T}}\frac{\partial T}{\partial x_{j}}\delta_{2j}$$

(13) $$\mu_{t}=C_{\mu }\frac{{\rho K}^{2}}{\varepsilon }$$

### 2.3. Overall Ventilation Effectiveness Equations

In addition to the average air temperature and the average concentration of CO_2_ in the interior of the space, calculated using the temperature and concentration fields obtained by the governing equations, it is important to quantify how the comfort temperature and the contaminant are distributed in the analyzed space. In this regard, Awbi [35] established that an efficient ventilation system is a result of good air quality and good thermal comfort and proposed Equation (14) to define overall ventilation effectiveness for air temperature distribution and Equation (15) to define the overall ventilation effectiveness for CO_2_ removal:

(14) $$E_{T}=\frac{T_{\mathrm{out}}-T_{\mathrm{in}}}{T_{A}-T_{\mathrm{in}}}$$

(15) $$E_{C}=\frac{C_{\mathrm{out}}-C_{\mathrm{in}}}{C_{A}-C_{\mathrm{in}}}$$

In the above equations, T_out_ and C_out_ are temperature and CO_2_ concentration at the outlet, respectively. T_in_ and C_in_ are, respectively, temperature and CO_2_ concentration at the inlet. T_A_ and C_A_ are, respectively, temperature and CO_2_ concentration averages inside the room.

### 2.4. Numerical Method

The governing equations are solved using the finite element method [36,37]. The scalar variables are calculated in the main mesh, while the velocity components are calculated in another mesh, which is twice finer than the main mesh. The discretization is carried out by means of an operator separation scheme that decouples the non-linearity of the Navier–Stokes equations. This scheme allows to solve the conservation equations by the following subproblems:

(16) $$\int_{\Omega }^{}\frac{\partial u_{i}}{\partial x_{i}}\psi d\Omega =0$$

(17) $$\int_{\Omega }^{}u_{j}\frac{\partial u_{i}}{\partial x_{j}}\psi d\Omega =\int_{\Omega }^{}P^{n}\frac{\partial \Psi }{\partial x_{i}}d\Omega$$

(18) $$\int_{\Omega }^{}u_{j}\frac{\partial T}{\partial x_{j}}\psi d\Omega +\int_{\Omega }^{}\frac{\lambda }{\rho \cdot C_{p}}\frac{\partial T}{\partial x_{j}}\frac{\partial \Psi }{\partial x_{j}}d\Omega -\int_{\Omega }^{}\frac{\mu_{t}}{\rho \cdot {\mathrm{Pr}}_{t}}\frac{\partial T}{\partial x_{j}}\frac{\partial \Psi }{\partial x_{i}}d\Omega =\int_{\Gamma }^{}T_{D}\Psi d\Gamma$$

(19) $$\int_{\Omega }^{}u_{j}\frac{\partial C}{\partial x_{j}}\psi d\Omega +\int_{\Omega }^{}D\frac{\partial C}{\partial x_{j}}\frac{\partial \Psi }{\partial x_{j}}d\Omega -\int_{\Omega }^{}\frac{\mu_{t}}{{\mathrm{Sc}}_{t}}\frac{\partial C}{\partial x_{j}}\frac{\partial \Psi }{\partial x_{i}}d\Omega =\int_{\Gamma }^{}C_{D}\Psi d\Gamma$$

(20) $$\int_{\Omega }^{}\left(\mu +\mu_{t}\right)\frac{\partial u_{i}}{\partial x_{j}}\frac{\partial \Psi }{\partial x_{j}}d\Omega =\int_{\Omega }^{}\frac{2}{3}{\rho K\delta }_{\mathrm{ij}}\Psi d\Omega +\int_{\Omega }^{}F_{i}\Psi d\Omega +\int_{\Gamma }^{}u_{i}{}_{D}\Psi d\Gamma$$

(21) $$\int_{\Omega }^{}u_{j}\frac{\partial K}{\partial x_{j}}\psi d\Omega +\int_{\Omega }^{}\left(\mu +\frac{\mu_{t}}{\sigma_{K}}\right)\frac{\partial K}{\partial x_{j}}\frac{\partial \Psi }{\partial x_{j}}d\Omega =\int_{\Omega }^{}P_{K}\psi d\Omega +\int_{\Omega }^{}G_{K}\psi d\Omega -\int_{\Omega }^{}\rho \epsilon \psi d\Omega$$

(22) $$\int_{\Omega }^{}u_{j}\frac{\partial \epsilon }{\partial x_{j}}\psi d\Omega +\int_{\Omega }^{}\left(\mu +\frac{\mu_{t}}{\sigma_{\varepsilon }}\right)\frac{\partial \epsilon }{\partial x_{j}}\frac{\partial \Psi }{\partial x_{j}}d\Omega \;\;=\int_{\Omega }^{}\frac{\epsilon }{K}C_{\epsilon 1}\left(P_{K}+C_{\epsilon 3}G_{K}\right)\psi d\Omega -\int_{\Omega }^{}\rho \frac{\epsilon }{K}C_{\epsilon 2}\epsilon \psi d\Omega$$

**Figure 3:**
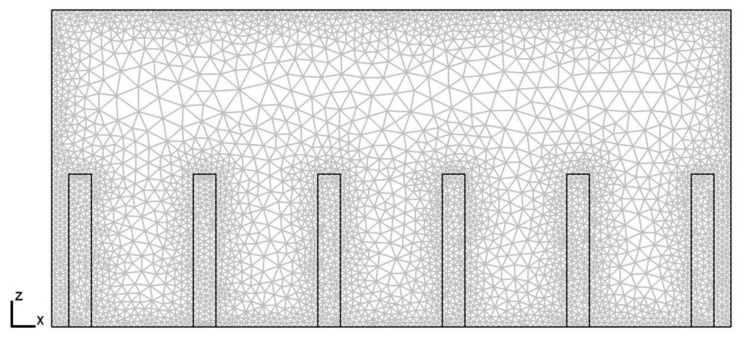
Cross-section of the computational mesh used in the simulations.

The appropriate mesh size is obtained by a mesh sensitivity study in which the behavior of the average temperature inside the classroom is analyzed for six different mesh sizes with the following node numbers: 255825, 350340, 462348, 550220, 650450, 751825, and 849246. Table 2 shows the behavior of average air temperature convergence for different cases. The maximum difference in the average air temperatures between the two finer meshes is 0.28 °C for case III, 6:30 p.m., C_s_ = 37,500 ppm, and Re = 15,000. The simulations were carried out by applying mesh refinement towards the walls in order to have greater precision in these regions (see Figure 3). The steady-state simulations were obtained when the consecutive values of each variable were less than 10^−6^. The spatial discretization was of the order of 0.01 m.

### 2.5. Validation

**Figure 4:**
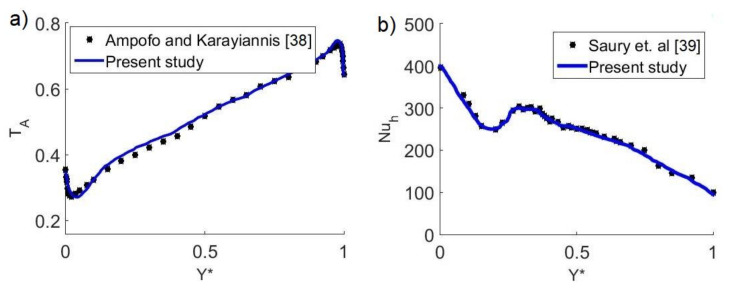
Validation of numerical results: (**a**) Comparison between mean temperatures for turbulent convection in an air-filled square cavity problem as reported by Ampofo and Karayiannis [38]. (**b**) Comparison of local Nusselt numbers along the hot wall for the turbulent convection problem in a differentially heated cavity as reported by Saury et al. [39].

To demonstrate precision and validate the obtained calculations, we solved the problem of turbulent natural convection in a square cavity with air. This turbulent natural convection problem was experimentally studied by Ampofo and Karayiannis [38]. In Figure 4a, the average temperature profiles in the transverse central plane of the cavity are compared for Rayleigh numbers of Ra = 1.58 × 10^9^. Additionally, the problem of turbulent flow was solved in a cavity with two differentially heated opposite vertical walls as proposed by Saury et al. [39]. In Figure 4b, the local Nusselt numbers in the hot wall are compared for Ra = 1.2 × 10^11^.

On the other hand, experimental measurements of the indoor air temperature of the classroom were also made. Table 3 shows comparisons between these values for Case III, 6:30 p.m., C_s_ = 37,500 ppm, Re = 15,000. The maximum error of the numerical results compared to the experimental results was 2.4%. These comparisons show that the numerical code used in this study can solve turbulent flow problems. The numerical code was developed using the Fortran programming language, in the thermofluid group of the Technological Institute of Veracruz. The numerical code has previously been used to solve various thermofluid problems [40,41,42,43].

## 3. Results and Discussion

### 3.1. Velocity Fields

**Figure 5:**
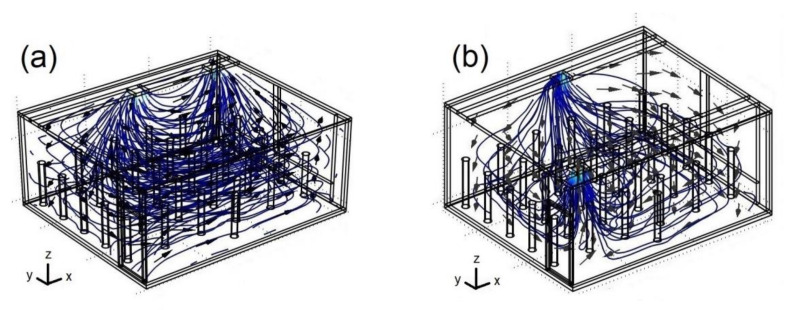
Velocity fields for: (**a**) case III; (**b**) case VI. Re = 15,000, 3:30 p.m. and C_s_ = 37,500 ppm.

Figure 5 presents velocity fields for two different extractor positions at 3:30 p.m. for Re = 15,000 and C_s_ = 37,500 ppm. Figure 5a shows the airflow patterns when the extractor is located in the corner formed by the wall with windows and the wall near the air-conditioning supply. In this case, the air-conditioning supply and the exhaust through the extractor are located on the same side. The flow patterns for this configuration show better air distribution inside the classroom, as the airflow circulates from the center of the south wall moving toward the east wall, then proceeds to the north wall, where it changes direction toward the west wall; and when it collides with this last wall, the airflow changes direction again toward the south wall, where it finally rises toward the extractor. Therefore, the air sweep for this case was the most effective since it covers all classroom areas. Figure 5b describes the behavior of the airflow inside the classroom when the extractor is located in the corner formed by the wall that contains the door and the wall opposite to the air-conditioning supply. In this case, the flow dynamics show that the air-conditioning stream is divided into two flows: one of them directed toward the west (hot) wall, which is the most intense, and the other, less intense stream moves toward the east (cold) wall. Subsequently, both streams move toward the north wall; the stream traveling along the east wall has a short trajectory since, when it collides with the north wall, it changes direction, moving toward the extractor in the ceiling. On the other hand, the stream that travels along the west wall has a long trajectory since, when it collides with the north wall, it travels along this entire wall until it reaches the east wall, where it rises toward the extractor. In this case, the air-conditioning supply and the exhaust of the extractor are located on opposite sides. As a result, the air sweep over the hot wall is more intense and has a longer trajectory, transporting a large amount of energy from the window into the classroom.

### 3.2. Temperatures Fields

**Figure 6:**
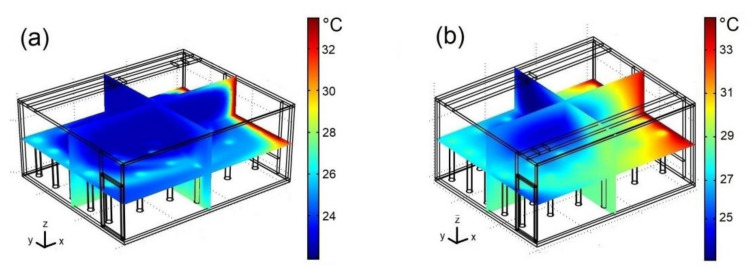
Temperature fields for: (**a**) case III; (**b**) case IV. Re = 15,000, 3:30 p.m. and C_s_ = 37,500 ppm.

To analyze the thermal behavior inside the classroom, Figure 6 describes the temperature fields for two different positions of the extractor at 3:30 p.m., Re = 15,000, and C_s_ = 37,500 ppm. Figure 6a presents the distribution of the air temperature inside the classroom when the extractor is located in the corner formed by the wall with windows and the wall near the air-conditioning system. A low temperature can be observed in the center of the classroom due to the cold air stream supplied by the air conditioning. The temperature tends to increase toward the side walls, where the windows and the door are located. However, this increase is greater on the west wall due to the heat flux transmitted by the windows, which are struck by the sun’s rays. The best distribution of cold air flow occurs when the extractor is on the same side as the air-conditioning system. On the other hand, the most adverse temperature field occurs when the extractor is located in the corner formed by the wall that contains the door and the wall opposite to the air-conditioning system (Figure 6b); in this case, significant temperature increases are observed even near the eastern wall. In addition, this configuration results in the largest reduction in the size of the cold region in the center of the classroom. In this configuration the extractor is placed on the side of the cold wall and away from the supply of the air-conditioning system.

According to these data, the case where the size of the cold central region is larger occurs when the extractor is located on the same side as the air-conditioning system and near the hot window, which is related to the fact that, in this case, air distribution is the most effective, covering all of the classroom zones. Furthermore, the case where the air temperature inside the classroom is kept less uniform and warmer in the central part occurs when the extractor is located on the opposite side of the air-conditioning system and close to the cold wall, which is related to the fact that, in this case, a sizeable part of the airflow follows a long trajectory along the west (hot) wall, transporting a large amount of energy into the classroom.

### 3.3. CO_2_ Concentration Fields

**Figure 7:**
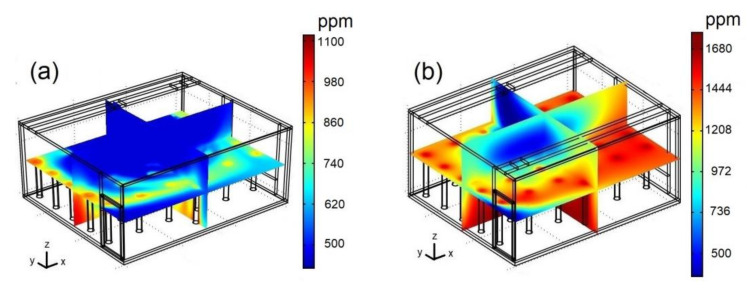
CO_2_ concentration fields for: (**a**) case III; (**b**) case IV. Re = 15,000, 3:30 p.m. and C_s_ = 37,500 ppm.

For a general idea of the spatial distribution of the pollutant inside the classroom, Figure 7 shows CO_2_ concentration fields for two different positions of the extractor at 3:30 p.m., Re = 15,000, and C_s_ = 37,500 ppm. The case where most of the classroom’s interior has low CO_2_ concentrations occurs when the extractor is located in the corner formed by the wall that contains the windows and the wall closest to the supply of the air-conditioning system (see Figure 7a). The worst configuration, with extensive high-CO_2_ regions, is when the extractor is located in the corner formed by the wall that contains the door and the wall opposite the air-conditioning system as shown in Figure 7b. In this case, significant increases in CO_2_ concentration are observed throughout the XY plane, while the only low-CO_2_ concentration region is the top center of the classroom.

In general, when the extractor is placed on the west wall (hot) side, CO_2_ concentrations decrease considerably, especially when the extractor is placed on the same side as the air-conditioning system (see Figure 7a). On the other hand, CO_2_ concentrations are very high in most of the classroom area when the extractor is placed on the east wall (cold) side, mainly when the extractor is placed opposite to the air-conditioning system (see Figure 7b).

### 3.4. Average Air Temperature

In the area of Veracruz, where the analyzed classroom is located, the climate is warm most of the year; accordingly, most people are used to the high-temperature environment. In this study, we considered the comfort temperature between 24.0 °C and 25.5 °C. When the temperature drops below the lower limit, most people start to feel cold. Furthermore, the greater the difference in temperature between indoor areas with air conditioning and outdoors, the greater the possibility of becoming sick due to sudden temperature changes.

**Figure 8:**
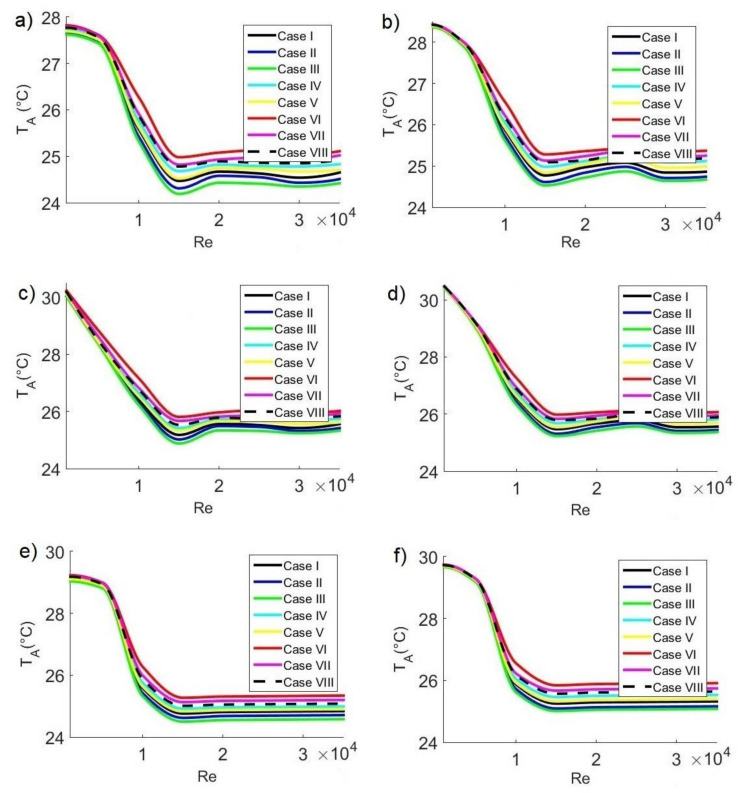
Average air temperature in the classroom as a function of Reynolds number at 11:30 a.m. with CO_2_ sources of (**a**) 35,000 ppm and (**b**) 42,500 ppm; at 3:30 p.m. with CO_2_ sources of (**c**) 35,000 ppm and (**d**) 42,500 ppm; and at 6:30 p.m. with CO_2_ sources of (**e**) 35,000 ppm and (**f**) 42,500 ppm.

Figure 8 shows the average temperatures T_A_ of air inside the classroom for three hours of the day as a function of the Reynolds number with different extractor locations and different concentrations C_s_ of pollutant sources. Figure 8a,b shows the cases at 11:30 a.m. for C_s_ = 35,000 ppm and C_s_ = 42,500 ppm, respectively. The minimum temperature values are T_A_= 24.19 °C for C_s_ = 35,000 ppm and T_A_ = 24.52 °C for C_s_ = 42,500 ppm. The maximum values are T_A_= 27.82 °C for C_s_ = 35,000 ppm and T_A_= 28.44 °C for C_s_ = 42,500 ppm.

Figure 8c,d shows the cases at 3:30 p.m. for C_s_ = 35,000 ppm and C_s_ = 42,500 ppm, respectively. The minimum temperature values are T_A_ = 24.88 °C for C_s_ = 35,000 ppm and T_A_ = 25.23 °C for C_s_ = 42,500 ppm. The maximum values are T_A_= 30.23 °C for C_s_ = 35,000 ppm and T_A_ = 30.48 °C for C_s_ = 42,500 ppm.

Figure 8e,f shows the cases at 6:30 p.m. for C_s_ = 35,000 ppm and C_s_ = 42,500 ppm, respectively. The minimum temperature values are T_A_ = 24.50 °C for C_s_ = 35,000 ppm and T_A_ = 25.01 °C for C_s_ = 42,500 ppm. The maximum values are T_A_ = 29.23 °C for C_s_ = 35,000 ppm and T_A_ = 29.74 °C for C_s_ = 42,500 ppm. All these minimum values are within the thermal comfort range and correspond to configuration III and Re = 15,000, while the maximum values occur for configuration IV and Re = 1000.

### 3.5. Overall Ventilation Effectiveness for Air Temperature Distribution

**Figure 9:**
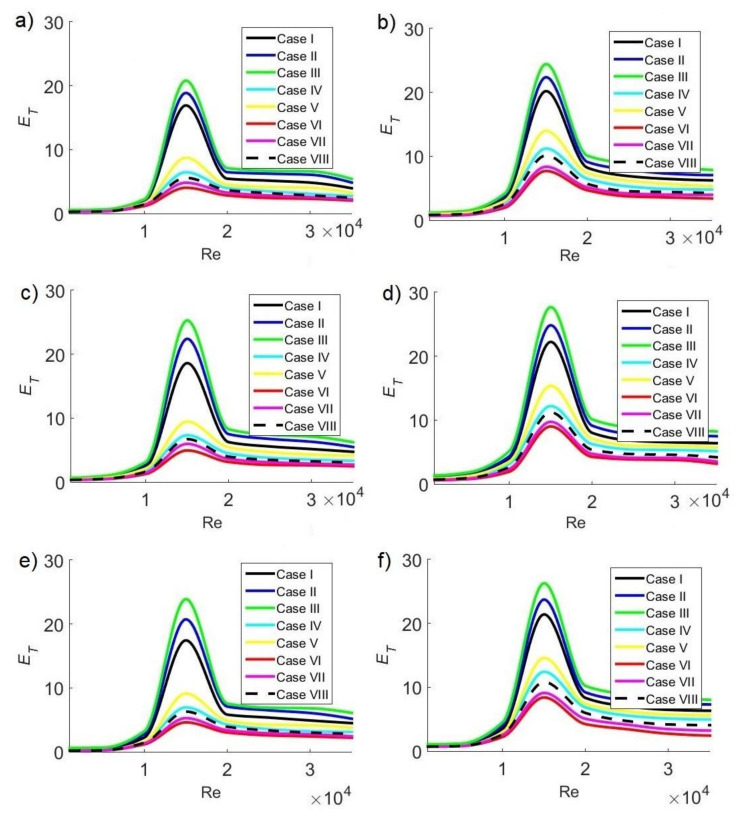
Global ventilation effectiveness for air temperature distribution in the classroom as a function of the Reynolds number at 11:30 a.m. with CO_2_ sources of (**a**) 35,000 ppm and (**b**) 42,500 ppm; at 3:30 p.m. with CO_2_ sources of (**c**) 35,000 ppm and (**d**) 42,500 ppm; and at 6:30 p.m. with CO_2_ sources of (**e**) 35,000 ppm and (**f**) 42,500 ppm.

Since the dynamics of airflow inside the classroom varies in each configuration, changing the spatial distribution of the temperature, it is necessary to evaluate overall ventilation effectiveness for temperature air temperature distribution E_T_. The higher the parameter value, the more homogeneous the interior temperature since the airflow improves heat removal. To analyze the effect of the Reynolds number on air temperature distribution inside the classroom, Figure 9 shows the E_T_ at different extractor positions and different concentration C_s_ of pollutant sources at 11:30 a.m., 3:30 p.m., and 6:30 p.m., respectively.

Figure 9a,b shows the cases at 11:30 a.m. for C_s_ = 35,000 ppm and C_s_ = 42,500 ppm, respectively. The maximum values of E_T_, where the most homogeneous air temperature distribution inside the classroom is guaranteed, are E_T_ = 20.78 and 24.43 for C_s_ = 35,000 and 42,500 ppm, respectively.

At 3:30 p.m., the maximum values are E_T_ = 25.28 (Figure 9c) and 27.68 (Figure 9d) for C_s_ = 35,000 and 42,500 ppm, respectively. Figure 9e,f shows the cases at 6:30 p.m. for C_s_ = 35,000 ppm and C_s_ = 42,500 ppm, respectively. The maximum values of E_T_ are 23.90 and 26.27 for C_s_ = 35,000 and 42,500 ppm, respectively. For the three times of the day considered, the maximum values occur for configuration III and Re = 15,000, which coincides with the condition where minimum T_A_ values are obtained.

The most favorable cases, where the temperature is within the range of thermal comfort, and the air temperature distribution is more homogeneous, occur for case III, which corresponds to the configuration where the air extractor is located on the same side as the air conditioning system’s air inlet and near the hot window. On the other hand, the configurations with the highest temperatures and the most unfavorable temperature distribution with low E_T_ values occur for case VI, when the extractor is located in the corner furthest from the air inlet of the air-conditioning system and close to the cold inner wall. Furthermore, the path of the air stream since supplied by the air-conditioning system to its exit through the extractor has a substantial effect on air temperature distribution: in all cases, the lowest average air temperature values and the maximum E_T_ values were observed for Re = 15,000. The highest values of average air temperature and the minimum E_T_ values were observed for Re = 1000. At low velocities, air temperature distribution is heterogeneous; however, as the velocity increases, the distribution becomes more homogeneous, reaching an optimal value when Re = 15,000.

### 3.6. Average CO_2_ Concentration

In general, safe limits of CO_2_ concentration are considered to be between 700 ppm and 1000 ppm. In this study, the safe limit of average CO_2_ concentration C_A_ inside the classroom was considered below 700 ppm, which guarantees a pleasant space where students can carry out their activities. Although analyzing the contagion of a disease requires more complex models that take into account the transport of exhaled droplets, the level of CO_2_ can be a practical measure in classrooms to reduce the risk of contagion since high levels of CO_2_ are an indication of agglomerations. Recent publications report that the concentration of CO_2_ in classrooms can help reduce the risk of transmission of contagious diseases, such as SARS-CoV-2, since low levels of CO_2_ indicate clean air [2,44,45,46,47].

**Figure 10:**
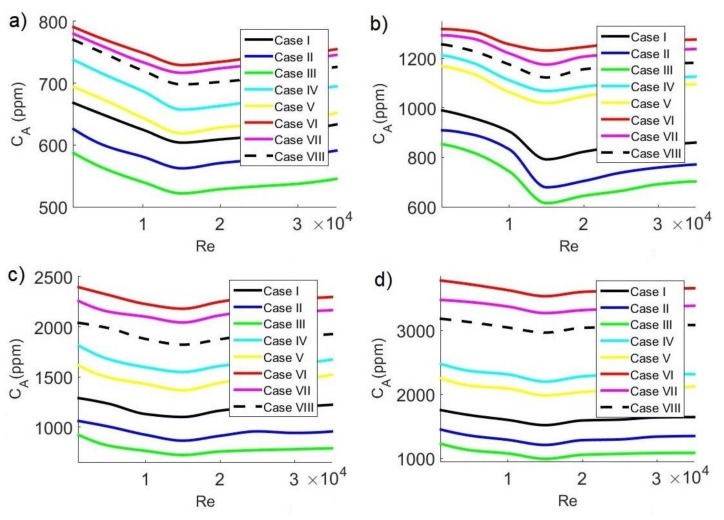
Average concentration of CO_2_ in the classroom as a function of Reynolds number at 11:30 a.m. with CO_2_ sources of: (**a**) 35,000 ppm; (**b**) 37,500 ppm; (**c**) 40,000 ppm; (**d**) 42,500 ppm.

**Figure 11:**
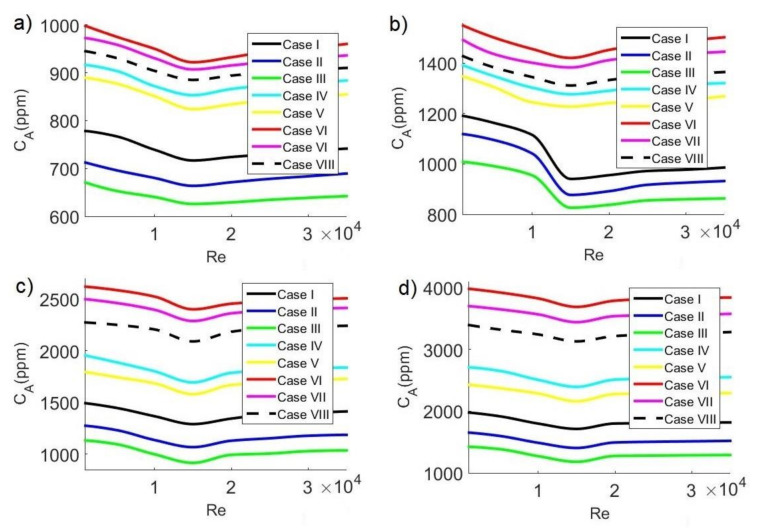
Average concentration of CO_2_ in the classroom as a function of Reynolds number at 3:30 p.m. with CO_2_ sources of: (**a**) 35,000 ppm; (**b**) 37,500 ppm; (**c**) 40,000 ppm; (**d**) 42,500 ppm.

**Figure 12:**
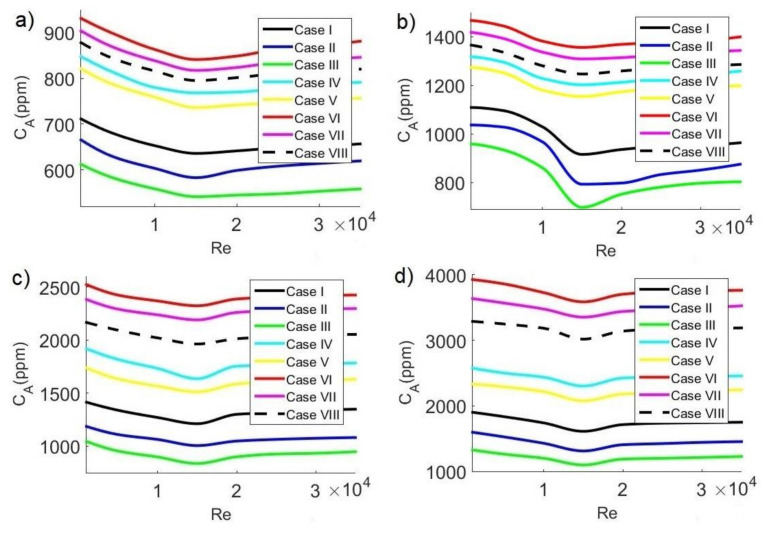
Average concentration of CO_2_ in the classroom as a function of Reynolds number at 6:30 p.m. with CO_2_ sources of: (**a**) 35,000 ppm; (**b**) 37,500 ppm; (**c**) 40,000 ppm; (**d**) 42,500 ppm.

Figure 10, Figure 11 and Figure 12 show average concentrations of C_A_ pollutant inside the classroom as a function of Reynolds number for different extractor locations and different concentrations C_s_ of pollutant sources. In general, it can be observed that the configurations where contaminant concentrations are lower occurred for case III, that is, when the air inlet and outlet are on the same side. On the other hand, the most unfavorable configurations occur in case VI, when the air inlet and outlet are on opposite sides. Air velocity has another significant effect on average pollutant concentration inside the classroom, observing in all cases: minimum average CO_2_ concentrations occurred for Re = 15,000, while the maximum values occurred for Re = 1000. This implies that, at low velocities, the release of CO_2_ is lower, while as the velocity increases, pollutant removal improves, reaching an optimal value when Re = 15,000. Concerning pollutant sources, we could observe that, as CO_2_ concentrations from sources increase, the average pollutant concentration value increases, too. Minimum C_A_ values occur with pollutant sources of 35,000 ppm, while maximum values occur with pollutant sources of 42,500 ppm.

The behavior of average concentration at 11:30 a.m. is shown in Figure 10. Figure 10a,b shows the cases for C_s_ = 35,000 ppm and C_s_ = 37,500 ppm, respectively. For C_s_ = 35,000 ppm, the minimum value is C_A_ = 521.77 ppm, while for C_s_ = 37500 ppm, the minimum value is C_A_ = 614.98 ppm, which occurs for configuration III and Re = 15,000. The safety limit (C_A_ < 700 ppm) is met for both conditions mentioned above, with a low risk of contagion by SARS-CoV-2. The most unfavorable configurations occur for case VI and Re = 1000, with maximum values of C_A_ = 790.82 ppm for C_s_ = 35,000 ppm and C_A_ = 1318.57 ppm for C_s_ = 37,500 ppm. On the other hand, Figure 10c,d shows the cases for C_s_ = 40,000 ppm and C_s_ = 42,500 ppm, respectively. For C_s_ = 40,000 ppm, the minimum value is C_A_ = 721.17 ppm, while for C_s_ = 42,500 ppm, the minimum value is C_A_ = 991.58 ppm, which occurs for configuration III and Re = 15,000. The safety limit is not met for the two conditions mentioned above since C_A_ > 700 ppm. The most adverse conditions occur for case VI and Re = 1000, reaching the maximum values of C_A_ = 2394.91 ppm for C_s_ = 40,000 ppm and C_A_ = 3778.51 ppm for C_s_ = 42,500 ppm.

Figure 11 shows the behavior of average concentration at 3:30 p.m. The case for C_s_ = 35,000 ppm is shown in Figure 11a, with a minimum value of C_A_ = 625.83 ppm, which occurs for configuration III and Re = 15,000; the average contaminant concentration for this condition indicates a low risk of contagion by SARS-CoV-2 since C_A_ < 700 ppm. The highest concentrations of CO_2_ occur for case VI and Re = 1000, reaching a maximum value of C_A_ = 998.18 ppm. On the other hand, Figure 11b–d shows the cases for C_s_ = 37,500 ppm, C_s_ = 40,000 ppm, and C_s_ = 42,500 ppm, respectively. For C_s_ = 37,500 ppm, the minimum value is C_A_ = 826.18 ppm; for C_s_ = 40,000 ppm, the minimum value is C_A_= 915.15 ppm, and for C_s_ = 42,500 ppm, the minimum value is C_A_ = 1180.29 ppm, which occur for configuration III and Re = 15,000. For the three conditions mentioned above, the maximum CO_2_ allowable value is unmet given that C_A_ > 700 ppm. The highest concentrations of the contaminant occur for case VI and Re = 1000, reaching maximum values of C_A_ = 1549.91 ppm for C_s_ = 37,500 ppm, C_A_ = 2619.13 ppm for C_s_ = 40,000 ppm, and C_A_ = 3983.77 ppm for C_s_ = 42,500 ppm.

The behavior of the average concentration at 6:30 p.m. are shown in Figure 12. Figure 12a,b shows the cases for C_s_ = 35,000 ppm and C_s_ = 37,500 ppm, respectively. For C_s_ = 35,000 ppm, the minimum value is C_A_ = 541.17 ppm, while for C_s_ = 37,500 ppm, the minimum value is C_A_ = 697.07 ppm (case III and Re = 15,000). The maximum allowable CO_2_ concentration (C_A_ < 700 ppm) is met for these two conditions, representing a low risk of contagion by SARS-CoV-2. The maximum values are C_A_ = 931.09 ppm for C_s_ = 35,000 ppm and C_A_ = 1467.41 ppm for C_s_ = 37,500 ppm (case VI and Re = 1000). On the other hand, Figure 12c,d shows the cases for C_s_ = 40,000 ppm and C_s_ = 42,500 ppm, respectively. For C_s_ = 40,000 ppm, the minimum value is C_A_ = 836.91 ppm, while for C_s_ = 42,500 ppm, the minimum value is C_A_ = 1098.12 ppm (configuration III and Re = 15,000). The maximum allowable pollutant concentration value is unmet in these two conditions since C_A_ > 700 ppm. The maximum values are C_A_ = 2520.95 ppm for C_s_ = 40,000 ppm and C_A_ = 3927.15 ppm for C_s_ = 42,500 ppm (case VI and Re = 1000).

### 3.7. Overall Ventilation Effectiveness for CO_2_ Removal

**Figure 13:**
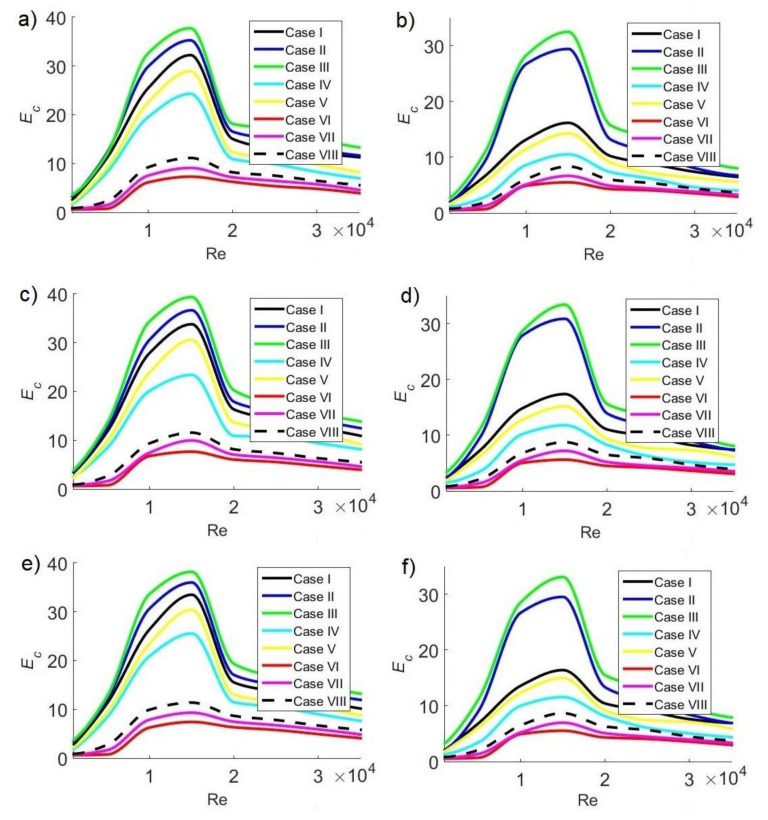
Overall ventilation effectiveness for CO_2_ removal in the classroom as a function of Reynolds number at 11:30 a.m. with CO_2_ sources of (**a**) 35,000 ppm and (**b**) 42,500 ppm; at 3:30 p.m. with CO_2_ sources of (**c**) 35,000 ppm and (**d**) 42,500 ppm; and at 6:30 p.m. with CO_2_ sources of (**e**) 35,000 ppm and (**f**) 42,500 ppm.

To analyze the effect of the Reynolds number on pollutant distribution inside the classroom, Figure 13 describes overall ventilation effectiveness E_c_ for CO_2_ removal at different extractor positions and different concentrations C_s_ of pollutant sources at 11:30 a.m., 3:30 p.m., and 6:30 p.m., respectively. High E_c_ indicates greater CO_2_ distribution uniformity.

Figure 13a,b shows the cases at 11:30 a.m. for C_s_ = 35,000 ppm and C_s_ = 42,500 ppm, respectively. Maximum E_c_ values where the most homogeneous pollutant distribution is guaranteed are E_c_ = 37.75 and 32.50 for C_s_ = 35,000 and 42,500 ppm, respectively. At 3:30 p.m., the maximum values of E_c_ are 39.36 (Figure 13c) and 33.43 (Figure 13d) for C_s_ = 35,000 and 42,500 ppm, respectively. Figure 13e,f shows the cases for C_s_ = 35,000 ppm and C_s_ = 42,500 ppm, respectively. The maximum E_c_ values are 38.21 and 33.11 for C_s_ = 35,000 and 42,500 ppm, respectively.

For the three hours of the day considered in the study, the most homogeneous pollutant distribution occurs for case III, which corresponds to the configuration where the extractor is located on the same side as the air inlet. On the other hand, the worst CO_2_ distribution, with low E_c_ values, occurs for case VI, when the extractor is placed on the opposite side of the air inlet. The flow pattern caused by the air-conditioning system’s injection and the effects of buoyancy near the hot window have noteworthy effects on pollutant distribution: in all cases, maximum values of E_c_ were observed for Re = 15,000, whereas minimum values occurred for Re = 1000. At low velocities, CO_2_ distribution is heterogeneous; however, as the velocity increases, the distribution becomes more homogeneous, reaching an optimal value when Re = 15,000.

### 3.8. Proposal to Reduce the Number of Students in Cases Where the Maximum Allowed Value of CO_2_ Is Exceeded

**Figure 14:**
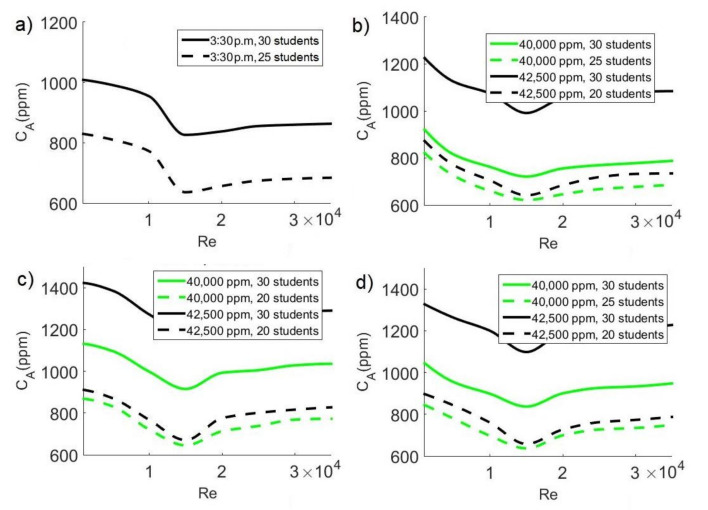
Reduction of the number of students to reach safe levels of CO_2_ concentration (C_A_ < 700 ppm) inside the classroom: (**a**) case III with Cs = 37500 ppm; (**b**) case III for 11:30 a.m.; (**c**) case III for 3:30 p.m.; (**d**) case III for 6:30 p.m.

Although, in most cases, average pollutant concentration can be reduced to less than 1000 ppm of CO_2_ by placing the ceiling extractor on the same side as the air-conditioning supply and close to the hot wall, in some configurations, this concentration remains above 700 ppm of CO_2_. Due to the above, in these cases, we propose a reduction in the number of students (see Figure 14) so that the concentration of the pollutant inside the space remains below the safe limit, thereby helping to avoid the adverse effects of agglomerations in closed areas, for example, the possibility of spreading contagious diseases, such as COVID-19, and any of chance for contamination.

When the CO_2_ source is 35,000 ppm, average pollutant concentration exceeds 700 ppm at the time of maximum heat flux through the window (3:30 p.m.). In this case, we propose reducing the number of students to 25 to achieve average CO_2_ concentrations below 700 ppm as shown in Figure 14a. This behavior is achieved in case III, when the extractor is located in the corner formed by the wall containing the windows and the wall near the air-conditioning system. On the other hand, the concentration described above occurs when Re = 15,000.

When CO_2_ sources are of 40,000 and 42,500 ppm, average pollutant concentration remains above the safe limit since C_A_ > 700 ppm; because of this, reducing the number of students is also proposed for these cases. At 11:30 a.m. (see Figure 14b) and 6:30 p.m. (see Figure 14d), with a pollutant source of 40,000 ppm, the maximum allowed value (C_A_ < 700 ppm) is attained by reducing the number of students to 25; for a pollutant source of 42,500 ppm, the number of students must be reduced to 20. At 3:30 p.m. (see Figure 14c), with pollutant sources of 40,000 and 42,500 ppm, it is also necessary to reduce the number of students to 20 to achieve average concentrations below 700 ppm. The minimum average concentration values occur when Re = 15,000 for case III, when the air inlet and outlet inside the classroom are located on the same side.

## 4. Conclusions

This study carried out a numerical analysis of thermal comfort and CO_2_ levels inside an air-conditioned classroom. The analysis considered the effects of a ceiling extractor position and Reynolds number inside the classroom for different concentration conditions of the pollutant sources and three different times of the day. The classroom has two closed windows through which it receives thermal loads due to the heating of the sun’s rays. Based on our results, the following conclusions can be highlighted:

1. The most favorable flow patterns for adequate classroom ventilation were observed when the air-conditioning supply and the extractor exhaust were located on the same side (case III) because the air sweep covered all areas inside the classroom. Moreover, in case III, the classroom remained at thermal comfort temperatures and had the lowest CO_2_ concentration levels.
2. The worst classroom ventilation arrangement occurred when the air-conditioning supply and the extractor exhaust were located on opposite sides (case VI) because the supplied cold air could not reach all of the regions in the classroom. In addition, in case VI, most of the classroom remained at high temperatures and presented the highest pollutant levels.
3. At all pollutant concentrations and the three hours of the day considered in the study, the lowest average temperatures inside the classroom occurred in case III when Re = 15,000. These average temperature values were within the range of thermal comfort. Maximum average temperatures correspond to case VI and Re = 1000. Average temperatures increased slightly when the concentration of pollutant sources increased.
4. The lowest average CO_2_ concentrations (i.e., best removal of pollutants) inside the classroom occurred in case III when Re = 15,000 for all concentrations of the pollutant sources and the three hours of the day considered in the study. However, these average concentration values were within the safe range of CO_2_ levels (<700 ppm) only at 11:30 a.m. with C_s_ = 35,000 ppm and C_s_ = 37,500 ppm, at 3:30 p.m. with C_s_ = 35,000 ppm, and at 6:30 p.m. with C_s_ = 35,000 ppm and C_s_ = 37,500 ppm. For the other cases, reducing the number of students to less than 30 is advisable. The highest average CO_2_ concentrations (i.e., worst removal of pollutants) occurred in case VI and Re = 1000.
5. To comply with the maximum allowable CO_2_ concentration value (<700 ppm), we propose to reduce the number of students from 30 to 25 at 11:30 a.m. with C_s_ = 40,000 ppm, at 3:30 p.m. with C_s_ = 37,500 ppm, and 6:30 p.m. with C_s_ = 40,000 ppm. On the other hand, at 11:30 a.m. with C_s_ = 42,500 ppm, at 3:30 p.m. with C_s_ = 40,000 ppm and C_s_ = 42,500 ppm, and 6:30 p.m. with C_s_ = 42,500 ppm, the number of students must be reduced from 30 to 20 students.
6. The proposed strategies can be used to prevent CO_2_ levels from exceeding the safe value of 700 ppm; in addition, thermal comfort and air quality are guaranteed, and the risk of contagion by COVID-19 in classrooms is reduced.

## Figures and Tables

**Table 1:** Properties of the air-CO_2_ mixture.

Cp (J/kg⋅K)	ρ (kg/m^3^)	µ (kg/m⋅s)	λ (W/m⋅K)	D (m^2^/s)
997.8	1.135	1.891 × 10^−5^	2.65 × 10^−2^	1.5 × 10^−5^

**Table 2:** Sensitivity analysis of the mesh for average air temperature inside the classroom.

Mesh Nodes	255825	350340	462348	550220	650450	751825	849246
Case III, 11:30 a.m., C_s_ = 35,000 ppm, Re = 15,000							
Ta (°C)	17.04	19.84	21.86	23.30	24.19	24.35	24.49
ΔT (°C)	-	2.8	2.02	1.44	0.89	0.16	0.14
Case III, 11:30 a.m., C_s_ = 37,500 ppm, Re = 15,000							
Ta (°C)	18.12	21.26	23.64	25.16	24.33	24.64	24.90
ΔT (°C)	-	3.14	2.38	1.52	0.83	0.31	0.26
Case III, 3:30 p.m., C_s_ = 35,000 ppm, Re = 15,000							
Ta (°C)	23.37	18.99	21.71	23.62	24.88	25.12	25.32
ΔT (°C)	-	4.83	2.72	1.91	2.26	0.24	0.20
Case III, 6:30 p.m., C_s_ = 37,500 ppm, Re = 15,000							
Ta (°C)	18.99	22.37	25.48	23.41	24.73	25.07	25.35
ΔT (°C)		3.38	3.11	2.07	1.32	0.34	0.28

**Table 3:** Comparisons of air temperatures inside the classroom, experimentally measured and numerically obtained.

Exp.	Num.	Error	Exp.	Num.	Error	Exp.	Num.	Error	Exp.	Num.	Error
T1 (°C)			T2 (°C)			T3 (°C)			T4 (°C)		
X = 5.5 m	Y = 0.5 m		X = 5.5 m	Y = 2.5 m		X = 5.5 m	Y = 4.5 m		X = 4.5 m	Y = 0.5 m	
24.52	24.07	1.8%	24.71	24.14	2.3%	24.77	24.39	1.5%	24.82	24.34	1.9%
T5 (°C)			T6 (°C)			T7 (°C)			T8 (°C)		
X = 4.5 m	Y = 2.5 m		X = 4.5 m	Y = 4.5 m		X = 3.0 m	Y = 0.5 m		X = 3.0 m	Y = 1.5 m	
23.18	22.85	1.4%	23.45	23.05	1.7%	23.68	23.15	2.2%	23.39	22.99	1.7%
T9 (°C)			T10 (°C)			T11 (°C)			T12 (°C)		
X = 3.0 m	Y = 3.5 m		X = 3.0 m	Y = 4.5 m		X = 1.5 m	Y = 0.5 m		X = 1.5 m	Y = 2.5 m	
22.84	22.72	0.5%	23.14	23.07	0.3%	22.93	23.31	1.6%	23.54	23.09	1.9%
T13 (°C)			T14 (°C)			T15 (°C)			T16 (°C)		
X = 1.5 m	Y = 4.5 m		X = 0.5 m	Y = 0.5 m		X = 0.5 m	Y = 2.5 m		X = 0.5 m	Y = 4.5 m	
23.67	23.48	0.8%	23.54	23.88	1.4	23.61	24.07	1.9	23.58	24.11	2.2%

## Data Availability

Not applicable.
